# Biological Activities of Etodolac‐Based Hydrazone, Thiazolidinone and Triazole Derivatives on Breast Cancer Cell Lines MCF‐7 and MDA‐MB‐231

**DOI:** 10.1002/jbt.70428

**Published:** 2025-08-11

**Authors:** Sevgi Kocyigit Sevinc, Pelin Çıkla‐Süzgün, Pinar Mega Tiber, Ş. Güniz Küçükgüzel, Oya Orun

**Affiliations:** ^1^ Department of Biophysics, Faculty of Medicine Marmara University Istanbul Turkey; ^2^ Department of Biophysics, Faculty of Medicine Kütahya Health Sciences University Kütahya Turkey; ^3^ Department of Pharmaceutical Chemistry, Faculty of Pharmacy Marmara University Istanbul Turkey; ^4^ Department of Pharmaceutical Chemistry, Faculty of Pharmacy Fenerbahce University Istanbul Turkey

**Keywords:** apoptosis, breast cancer, cell viability, cyclooxygenase, etodolac, prostaglandin E2

## Abstract

In this study, several etodolac‐based hydrazone, thiazolidinone, and triazole derivatives that we synthesized and characterized in our earlier research were tested against the hormone‐responsive breast cell line MCF‐7 and the triple‐negative MDA‐MB‐231, as well as the murine origin fibroblast cell line L‐929, at varying doses for their effects on cell viability and toxicity and for their inhibitory activity on cyclooxygenase‐2 (COX‐2)/prostaglandin E2 (PGE2) formation. Cell viability and apoptosis tests were utilized to assess the anti‐cancer potential of etodolac and its derivatives after the cells were exposed to varied concentrations of synthesized compounds for three different time periods. ELISA and Western blot methods were used to detect protein levels. All synthesized compounds demonstrated higher anti‐cancer activity at significantly lower doses compared to etodolac (half‐maximal inhibitory concentration [IC50] of 0‐50 µM range in derivatives versus 0.5−1 mM range in etodolac). Except for SGK 242, which had a major toxic effect on all cells, the chemicals SGK 206 and SGK 217 had a twice‐less impact on control murine L‐929 fibroblasts. Similar to proliferation, low concentrations of SGK 206 and SGK 217 (25−50 µM) significantly induced apoptosis in breast cancer cells but not in normal cells. Additionally, they inhibited COX‐2 protein expression at 50 µM, and SGK 206 inhibited PGE2 release more effectively than etodolac in cancer cells. The results of this study suggest that, in comparison to a healthy control group, the thiazolidinone derivative SGK 206 and the thiazolidinone derivative SGK 217 are more effective than etodolac when it comes to the breast cancer cell lines MCF‐7 and MDA‐MB‐231. SGK 206 exhibits a low IC50 value, a distinct dose–response relationship, and strong apoptotic effects, particularly on MDA‐MB‐231 cells.

## Graphical abstract

Both the etodolac hydrazone derivative, SGK 206, and the thiazolidinone derivative, SGK 217, were found to be more effective than etodolac in MCF‐7 and MDA‐MB‐231 breast cancer cell types. In particular, SGK 206 has the potential to be a compelling novel therapeutic candidate that drives future investigation.

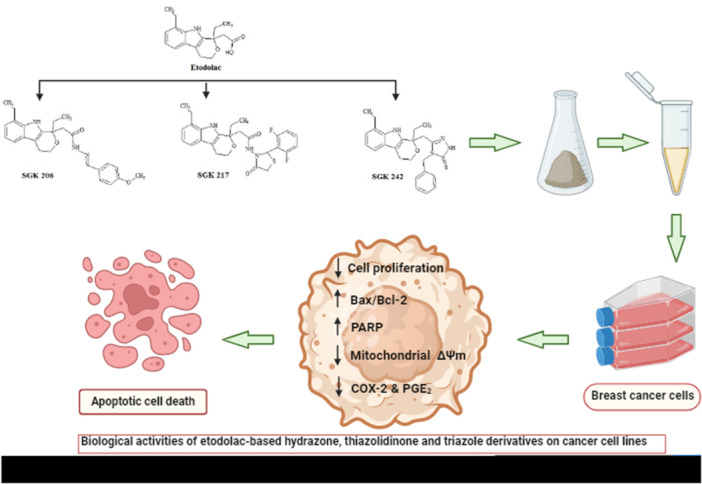

## 1 Introduction

Etodolac [2‐(1,8‐diethyl‐1,3,4‐tetrahydropyrano [3,4‐b] indol‐1‐yl) acetic acid] is a non‐steroidal anti‐inflammatory drug (NSAID) with analgesic and antipyretic properties [1, 2, 3, 4]. It has been approved by the US Food and Drug Administration (FDA) and has been available in the market for several years, primarily used for the treatment of pain associated with inflammation and conditions such as rheumatoid arthritis and osteoarthritis [5, 6, 7, 8]. Etodolac exerts its therapeutic effects by inhibiting prostaglandin synthesis in inflamed tissues, thereby reducing and preventing the sensitization of pain receptors to inflammatory mediators such as histamine, serotonin, and bradykinin.

Etodolac suppresses the activity of cyclooxygenase enzymes (COX), which play a critical role in the biosynthesis of prostanoids from arachidonic acid, a 20‐carbon unsaturated fatty acid present in cell membranes. COX‐1 is constitutively expressed and found in various mammalian cells, whereas COX‐2 is typically undetectable in most cells and is only activated in response to inflammatory stimuli by macrophages and other cell types. Recent research suggests that, in addition to their role in inflammation, COX‐2 and prostaglandin E2 (PGE2) are also integral to the initiation and progression of various types of tumors [9, 10, 11, 12]. Epidemiological studies and clinical outcomes supported this theory, demonstrating that NSAIDs may prevent cancer development by suppressing COX‐2 activity [13, 14, 15].

Etodolac exhibits a thousand‐fold greater selectivity for COX‐2 over COX‐1 and is three times more selective for COX‐2 than meloxicam, another COX‐2 specific inhibitor [16]. Therefore, the antitumor activity of etodolac has been extensively investigated in various malignancies, including those originating in the urogenital system, Burkitt′s lymphoma, gastric cancer, colorectal cancer, multiple myeloma, and hepatocellular carcinoma [17, 18, 19].

In this study, etodolac served as the starting material, and its hydrazide‐hydrazone, 4‐thiazolidinone,and 1,2,4‐triazole derivatives (SGK 206, SGK 217, and SGK 242) were synthesized in Prof. Kucukguzel′s laboratory, as previously reported [20, 21, 22]. The cytotoxic and apoptotic effects of various derivatives of these three moieties have been previously investigated across multiple cancer types, both individually and in combination with potential anticancer agents [23, 24, 25, 26, 27, 28, 29, 30].

The hydrazone functional group can interact with biological molecules, resulting in the inhibition of cell proliferation and the induction of cell death through various mechanisms [31, 32, 33, 34, 35]. Additionally, derivatives incorporating 4‐thiazolidinone and 1,2,4‐triazole moieties have been identified as highly effective agents and significant targets in cancer therapy [36, 37, 38, 39]. Compared to the parent compound, the majority of these hybrid derivatives exhibit enhanced selectivity and anticancer activity [30, 37, 38, 40]. Intense study in recent years revealed actions involving different cellular responses such as inhibition of AKT, ERK, or p38 MAPK pathways, cell cycle arrests at S‐, G1‐, or G2/M phases, and induction of mitochondrial apoptotic processes [38, 39, 40, 41]. In addition to the above‐listed functions, studies conducted on MCF‐7 breast cancer cell lines with hydrazone derivatives have demonstrated their potential to block the epidermal growth factor receptor (EGFR) [42, 43]. Thiazole‐containing drugs have also been reported as effective protein kinase inhibitors, as the nitrogen and sulfur atoms in the thiazole ring possess the ability to form hydrogen bonds with the active sites of various protein kinases, including EGFR. Human epidermal growth factor receptor 2 (HER‐2), a member of the EGFR family, is well known for its therapeutic significance in breast cancer treatment. Because it may form weak chemical interactions with the environment, the triazole moiety has the useful capacity to increase the solubility of molecules. This characteristic makes it possible to create more advantageous medications with greater biological activity and reduced toxicity [44, 45, 46, 47]. Furthermore, both thiazole and triazole derivatives have been reported for their COX‐2 inhibitory activity. The effects of ibuprofen′s 1,2,4‐triazole derivatives on the enzymes COX1/2 and PGE2‐synthesis have been documented [48]. Similarly, two recent studies conducted by our group investigated the hydrazide‐hydrazone derivatives of etodolac in leukemia and prostate cancer, revealing their potential as COX‐2 and HER‐2 inhibitors, along with their antiproliferative and pro‐apoptotic activities [48, 49, 50].

In a pre‐screening assay involving 60 human tumor cell lines, the compounds exhibited substantial levels of growth inhibition (95%−100%) and lethality (5%−20%) at a 10 µM dosage in breast cancer cell lines that were selected for further investigation. Following full characterization, their biological effects were evaluated in breast cancer cell lines and control fibroblasts, including assessments of cytotoxicity, apoptotic induction, and modulation of the COX‐2/PGE2 pathway. In this context, the present study aims to identify novel compounds that, compared to etodolac, exhibit enhanced anticancer activity and selectivity in vitro while maintaining biological tolerance.

## 2 Materials and Methods

### 2.1 Cell Culture

MCF‐7, MDA‐MB‐231, and L‐929 mouse fibroblast cell lines were obtained from the American Type Culture Collection (ATCC, Manassas, Virginia, USA). MCF‐7 is a hormone‐responsive, differentiated mammary epithelium cell line with an ER+, PR+, and HER2‐ pattern (ATCC, HTB‐22). MDA‐MB‐231 is a triple‐negative differentiated mammary epithelium cell line (ATCC, HTB‐26). The L‐929 mouse fibroblast cell line is derived from the normal subcutaneous connective tissue of a mouse and is commonly used in cytotoxicity assays (ATCC, CCL‐1). All cell lines were cultured in DMEM medium (Dulbecco's Modified Eagle Medium, Gibco), supplemented with 10% fetal bovine serum (Gibco), 1% glutamine, and penicillin/streptomycin (Gibco) in a humidified atmosphere containing 5% CO_2_ at 37°C. Cell viability was tested using Trypan blue staining [51, 52].

### 2.2 In Vitro Cell Viability Assay

The MTT [3‐(4,5‐dimethylthiazol‐2‐yl)−2,5‐diphenyltetrazolium bromide] assay is a colorimetric assay for determining cell metabolic activity. In the MTT assay, the yellow dye MTT that dissolves in water is changed by mitochondrial reductase into a purple formazan that is non‐soluble. The water‐insoluble formazan produced is solubilized in a solubilization buffer, and absorbances are detected.

Using an MTT test, we assessed the cytotoxic and anti‐proliferative properties of etodolac and its derivatives (SGK 206, SGK 217, and SGK 242) in our setup in compliance with the supplier's instructions (Cell Proliferation Kit I, Roche 11 465 007 001). In 96‐well plates, the cells were cultivated at a density of 5 × 10^3^ cells/well using 100 µL medium. After being dissolved in one milliliter of DMSO, the stock solutions for etodolac, SGK 206, SGK 217, and SGK 242 had final yields of 70, 48, 43, and 47 mM. The cells were cultured for 24, 48, and 72 h after being exposed to different concentrations of etodolac and its derivatives (10, 25, 50, 75, and 100 µM). 10 μL of the MTT labeling reagent (final concentration 0.5 mg/mL) were added to each well at the end of the incubation periods, and the wells were incubated for an additional 4 h. Next, each well received 100 μL of solubilization buffer (10% SDS in 0.01 M HCl) and was overnight incubated, and the absorbance of formazan products was evaluated at 550 nm using a multi‐mode microplate reader (Synergy H1, Biotech Instruments Inc., Vermont, USA). The results were normalized relative to the absorbances of calculated percent concentrations of DMSO. All tests involved three replicates with three independent experiments.

### 2.3 Annexin‐V/Propidium Iodide (PI) Staining

To assess apoptosis and cell death, cells were stained with Annexin V‐PI dyes (Tali Apoptosis Kit‐Annexin V Alexa Fluor 488 and PI‐, Molecular Probes A10788, Invitrogen) and quantified using Tali Image‐Based Cytometer (Invitrogen) and fluorescence microscopy (Olympus CKX41 Inverted Phase Contrast Fluorescence Microscope furbished with reflected fluorescence illuminator CKX‐RFA).

### 2.4 Mitochondrial Membrane Potential (MMP) Assay

Apoptosis was also investigated by evaluating mitochondrial membrane potential changes (MMP assay). MCF‐7, MDA‐MB‐231, and L‐929 cells were stained with JC‐1 dye according to the manufacturer's protocols (JC‐1 MMP Assay Kit, KA1324, Abnova). The apoptotic to healthy cell ratio was calculated using absorbances collected from two different fluorescence channels (535/595 for healthy cells, 485/535 for apoptotic cells) (Synergy H1, Biotech Instruments Inc., Vermont, USA).

### 2.5 Determination of PGE2 Level

PGE2 ELISA kit (ENZO, ADI‐900‐001) was used to determine prostaglandin synthesis levels. MCF‐7 and MDA‐MB‐231 breast cancer cells were cultured at a density of 1 × 10^6^ cells/well in a 6‐well culture dish. After 24 h, cells were incubated with different concentrations of DUP‐697 (COX‐2 inhibitor, Cayman Chemical COX activity assay kit, item ♯760158), etodolac (500 µM), and SGK 206 (10, 25, and 50 µM). Following 24 h of incubation, the medium was replaced with a fresh one, 10 µM arachidonic acid was added, and it was further incubated for 1 h. The PGE2 levels were determined according to the kit protocol.

### 2.6 Western Blot

The cell lines treated with 50 and 75 µM SGK 206 and SGK 217 and 500 µM etodolac for 24 h were lysed with x1 RIPA buffer (50 mM Tris‐HCl, pH 8.0, 150 mM NaCl, 1% NP40, 0.5% Na‐deoxycholate, 0.1% SDS) containing protease inhibitor cocktail (Complete, EDTA‐free Protease Inhibitor Cocktail Tablets, Roche) and 10 mM sodium fluoride, a serine/threonine phosphatase inhibitor (Santa Cruz, SC‐24988B). Total protein levels were measured using the Take3 Multi‐Volume Plate (Synergy H1, BioTek Instruments Inc., Vermont, USA). In accordance with the classical protocol [53], transferred proteins were exposed to the following primary antibodies: Beta‐actin (NB600‐501, Novus Biologicals), Bax (SPM336, NBP2‐32809), Bcl‐2 (100/D5, NBP2‐15200), cleaved PARP (194C1439, NBP2‐27335), and COX‐2 (D‐12:sc‐166475, Santa Cruz). The bands were visualized with chemiluminescent HRP substrates (Western Bright ECL‐Advansta, K‐12045‐050). Using a chemiluminescence imaging system (Celvin S, BioStep), the relative quantification was done based on the level of β‐actin proteins.

### 2.7 Statistical Analysis

All statistical evaluations, including *t*‐tests and IC50 values, were calculated using the GraphPad Prism software tool (v6.0, GraphPad Software Inc., San Diego, USA). Statistical significance was defined as *p* ˂ 0.05 (*p < 0.05, **p < 0.01, ***p < 0.001, ****p < 0.0001).

## 3 Results and Discussion

### 3.1 Synthetic Procedures

**Figure 1:**
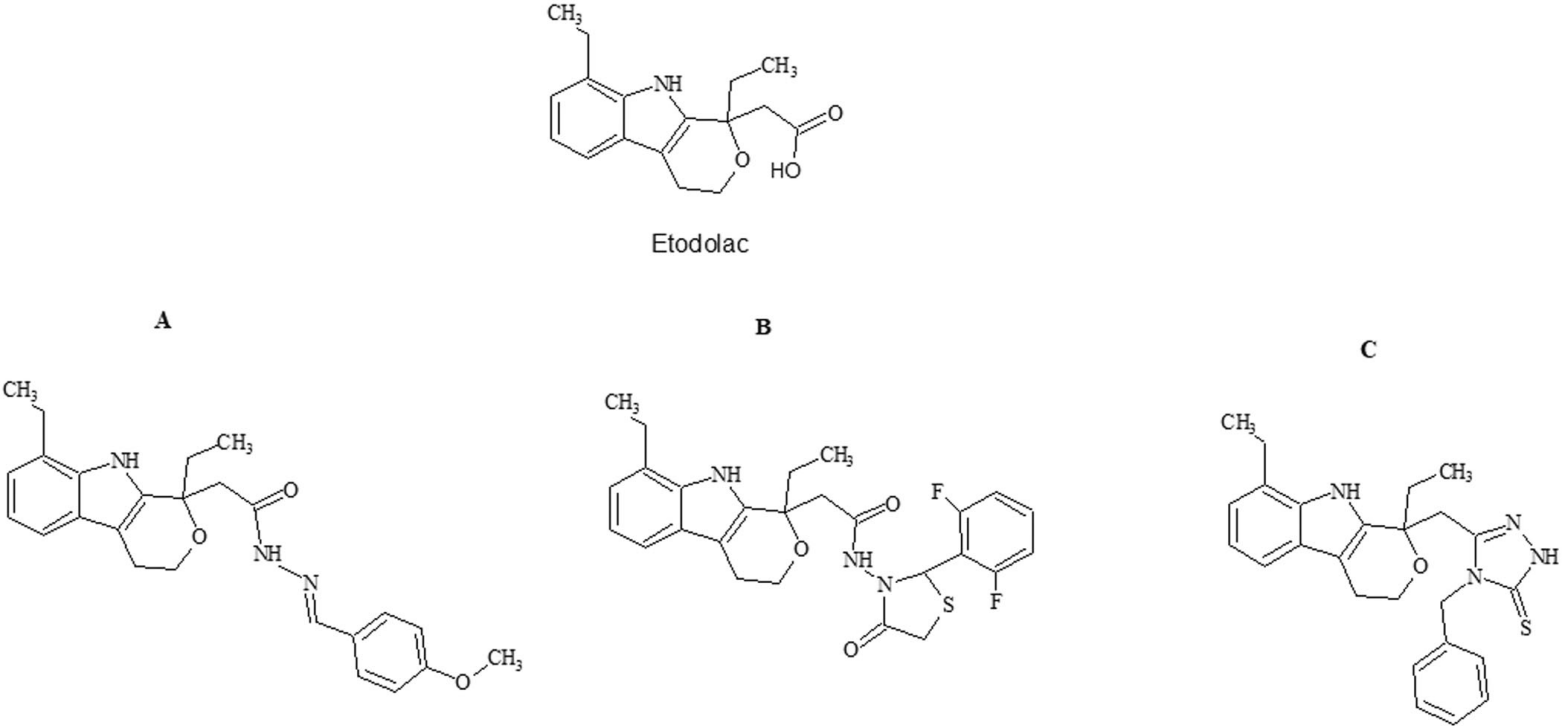
Synthesized derivatives of etodolac. (A) 2‐(1,8‐Diethyl‐1,3,4,9‐tetrahydropyrano[3,4‐b] indole‐1‐yl) acetic acid[(4‐methoxyphenyl)methylene]acetohydrazide [SGK 206]. (B) 3‐(2‐(1,8‐Diethyl‐1,3,4,9‐tetrahydropyrano[3,4‐b]indole‐1‐yl)acetylhydrazono)‐2‐(2,6‐difluorophenyl)‐4‐thiazolidinone [SGK 217]. (C) 5‐[(1,8‐Diethyl‐1,3,4,9‐tetrahydropyrano[3,4‐b]indole‐1‐yl)methyl]‐4‐benzyl‐2,4‐dihydro‐3H‐1,2,4‐triazole‐3‐thione [SGK 242].

In this study, (R, S)−2‐[1,8‐Diethyl‐1,3,4‐tetrahydrapyrano[3,4‐b] indole‐1‐yl]acetic acid (etodolac) was chosen as the initial molecule for the synthesis of hydrazide‐hydrazone (SGK 206), 4‐thiazolidinone (SGK 217), and 1,2,4‐triazole (SGK 242) derivatives (Figure 1A−C). Several derivatives of etodolac, combined with hydrazone [20, 21, 35, 49, 54, 55, 56, 57], including the chemicals of this study, were previously examined in a pre‐screening test against 60 cancer cell lines for a single dose (10^−5 ^M) [20, 21, 22]. The purity of the synthesized compounds was checked by thin‐layer chromatography (TLC) and microanalysis (CHNS). The structures of the obtained compounds were determined on the basis of FT‐IR, 1HNMR, 13C‐NMR, and HR‐MS spectral data, and all of the compounds have satisfactory analyses for their proposed structures. Based on the outcomes of these studies, here we set out to characterize three interesting compounds for their growth inhibitory and apoptotic effects: hydrazone (SGK 206), 4‐thiazolidinone (SGK 217), and 1,2,4‐triazole (SGK 242) derivatives of etodolac. The cytotoxicity and apoptotic properties of synthesized derivatives were thoroughly investigated for three time points, for different concentrations, in two breast cancer cell lines, MCF‐7 and MDA‐MB‐231, and non‐cancer control fibroblast cells L‐929.

### 3.2 Antiproliferative Activity of Compounds

Anti‐proliferative activities of etodolac and its derivatives were tested with the MTT assay. Etodolac was not very effective at low concentrations (0–100 μM), even after long incubation periods (48 and 72 h). The viability of the cells was reduced only at high concentrations (> 0.2−0.5 mM), and cytotoxicity was similar across all cell lines, including healthy controls, as previously reported [58]. The effective dose of etodolac on cancer cells displays a wide variety among the studies [59, 60, 61]. Early acceptance of etodolac as a medication is most likely based on its modest effects at relatively high doses, low toxicity, and side effect profile.

**Figure 2:**
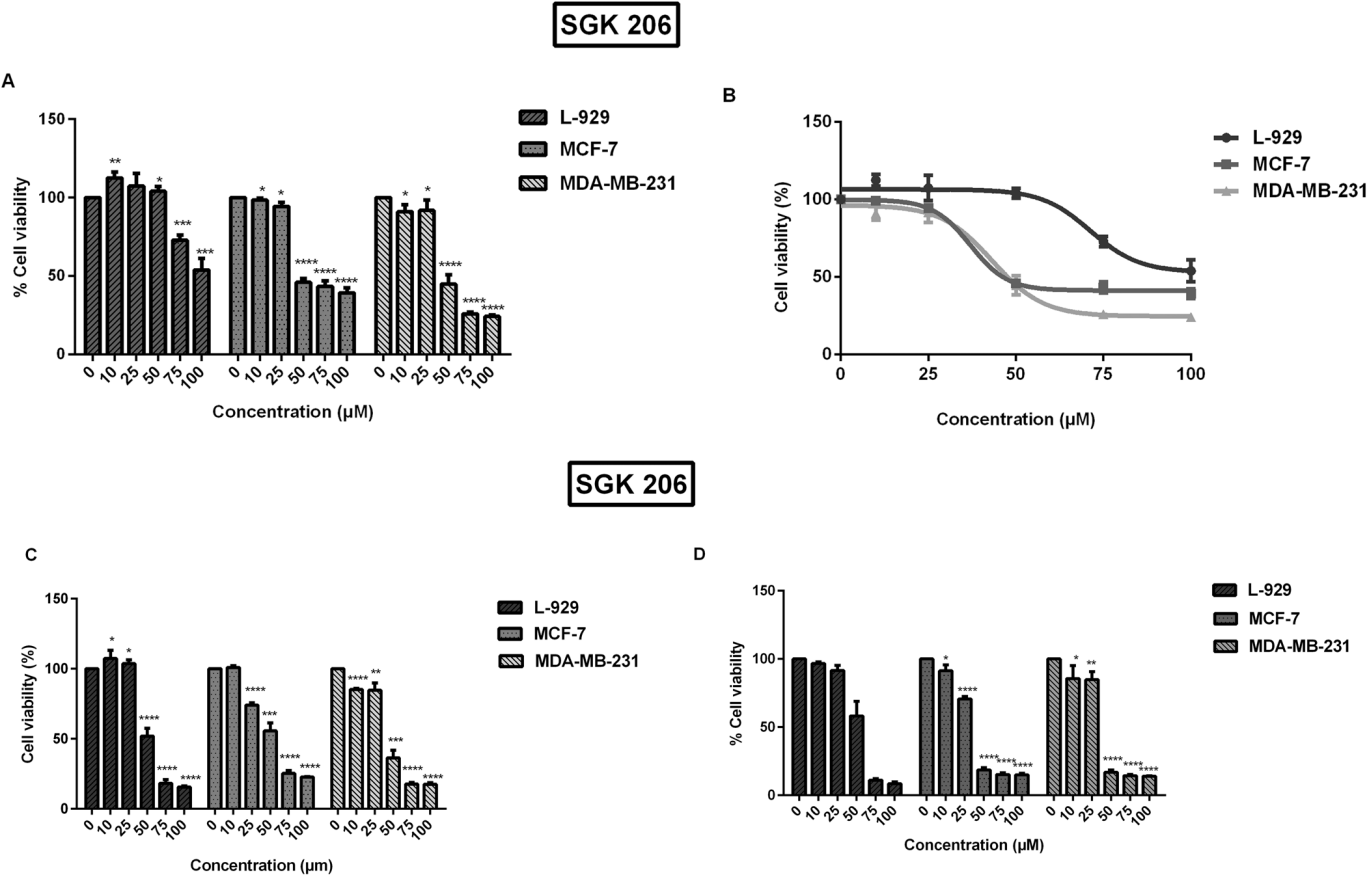
Cell viability changes due to SGK 206 activity. Viable cell numbers as a percent of control and corresponding dose–response curves at the end of (A, B) 24 h, (C, D) 48 h, and 72 h incubation periods. (*p < 0.05, **p < 0.01, ***p < 0.001, ****p < 0.0001).

On the other hand, the application of a hydrazide derivative, SGK 206, at low dosages (0−50 μM) remarkably decreased the viability of cancer cells, and the effect was substantially lower in fibroblast controls. In both breast cancer cell lines, the cytotoxic effect was strong, particularly at a 50 µM concentration, and significant at the *p* ˂ 0.0001 level. Additionally, viability loss was concentration‐dependent. In both cancer cell lines, SGK 206 elicited a clear dose‐dependent response, with IC50 values at the end of 24 h noted as 37, 43, and 71 µM for MCF‐7, MDA‐MB‐231, and L‐929 cells, respectively (Figure 2A,B). Treatment in longer periods further reduced IC50 values to 34, 38, and 47 µM for 48 h, as 26, 36, and 49 µM for 72 h in MCF‐7, MDA‐MB‐231, and L‐929 cells, respectively (Figure 2C,D).

**Figure 3:**
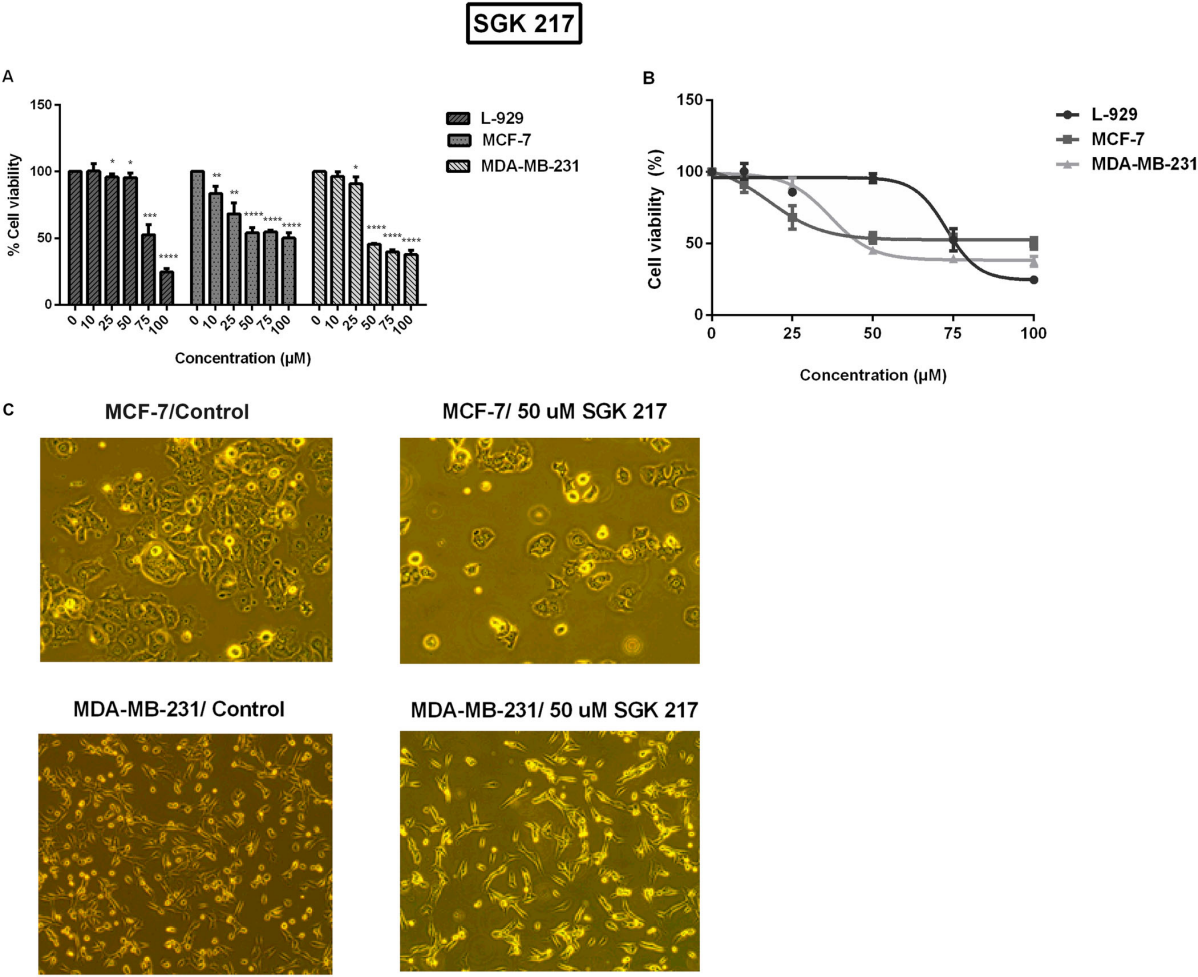
Cell viability changes due to SGK 217 activity. Viable cell numbers as a percent of control and corresponding dose–response curves at the end of (A, B) 24 h, (C) microscopic images of cells incubated with 50 µm SGK 217 for 24 h. (*p < 0.05, **p < 0.01, ***p < 0.001, ****p < 0.0001).

Similar to SGK 206, treatment with low doses of SGK 217 (0–50 µM) drastically decreased the viability of MCF‐7 and MDA‐MB‐231 cells while having a reduced effect on L‐929 fibroblast cells. At the end of a 24‐h treatment period, the IC50 values of SGK 217 in MCF‐7, MDA‐MB‐231, and L‐929 cells were found to be 18, 36, and 72 µM, respectively. The concentration‐dependent increase in cell toxicity was statistically significant at the *p* < 0.0001 level (Figure 3A). This compound showed a sigmoidal dose–response curve, especially in MDA‐MB‐231 cells (Figure 3B). The anti‐proliferative effect of 50 µM SGK 217 was also visualized with light microscopy (Figure 3C). The SGK 242 compound showed severe toxicity on all cell lines (MCF‐7, MDA‐MB‐231, and L‐929) at just 25 µM and significantly reduced cell viability. The toxicity was higher in the control L‐929 fibroblast cell line, and the response was not dose‐dependent (data not shown).

Tables 1 and 2 provide a summary of the selectivity indices and IC50 values calculated from viability experiments. Our compounds′ selectivity indices ranged from 1.5 to 2. When compared to L‐929 control cells, the tested drugs showed a modest level of selectivity towards cancer cells. The selectivity of SGK 206 for MCF‐7 and MDA‐MB‐231 cells against L‐929 was 1.93 and 1.65, respectively, after 24 h, as indicated by the IC50 values. This impact has diminished with time. Like SGK 206, SGK 217 also showed good selectivity against MCF‐7 and MDA‐MB‐231 cancer cells in a 24‐h period (SI: 4.0 and 2.0, respectively), suggesting that it may be a novel anticancer medication.

**Table 1:** The half‐maximum inhibitory concentrations (IC50) for the synthetic compounds of the study.

IC50 (µM)				
Compound	Time (h)	MCF‐7	MDA‐MB‐231	L‐929
Etodolac	24	750 ± 4.2	740 ± 1.65	735 ± 3.7
	48	731 ± 1.37	699 ± 4.61	750.8 ± 9.5
	72	555 ± 2.42	578 ± 3.65	622 ± 4.7
SGK 206	24	37 ± 1.74	43.2 ± 1.94	71.27 ± 2.92
	48	34 ± 5.7	38 ± 2.3	47.91 ± 1.2
	72	26 ± 0.66	36 ± 3.19	49.73 ± 1.64
SGK 217	24	18 ± 3.4	36 ± 1.6	72 ± 3.2
	48	12.2 ± 2.0	13.8 ± 1.6	29.6 ± 0.94
	72	26.7 ± 4.8	14.6 ± 1.4	26.3 ± 0.112
SGK 242	24	22 ± 0.1	31 ± 2.5	38 ± 8.05
	48	12 ± 3.3	24 ± 1.2	44.2 ± 2.45
	72	n.d.	n.d.	n.d.

**Table 2:** The selectivity indices (SI) for the synthetic compounds of the study, in comparison to the L‐929 cell line.

Selectivity indices	MCF‐7	MDA‐MB‐231	MCF‐7	MDA‐MB‐231	MCF‐7	MDA‐MB‐231
	SGK 206	SGK 206	SGK 217	SGK 217	SGK 242	SGK 242
24	1.93	1.65	4.0	2.0	1.7	1.2
48	1.4	1.26	2.4	2.1	3.6	1.8
72	1.91	1.38	1	1.8	n.d.	n.d.

As an FDA‐approved anti‐inflammatory agent, etodolac is a racemic blend of S‐ and R‐enantiomers. Strangely, the R‐enantiomer SDX‐101 was shown to have antitumor activity in chronic lymphocytic leukemia, though it has no COX inhibitory activity [63]. As depicted in Table 3, R‐enantiomer SDX‐101 and an indole‐pyran analog of etodolac SDX‐308 had anti‐proliferative activity in vitro in multiple myeloma cell lines, with IC50 values of 490 and 23.8 μM, respectively (Table 3). Huang et al. [64] investigated the cytotoxic effects of celecoxib on MCF‐7 and MDA‐MB‐231 monolayer cultures, reporting IC50 values of 40.05 and 89.05 µM, respectively. Notably, the SGK 206 and SGK 217 compounds used in this study exhibited greater cytotoxicity than celecoxib at lower concentrations in both cell lines (for MCF‐7, IC50s for SGK 206 is 37 µM and for SGK 217 it is 18 µM). Similarly, compared to other COX‐2 inhibitors, IC50 values from viability assays were lower than the reported values for chlorambucil, ibuprofen, naproxen, and indomethacin in the MCF‐7 cell line [67].

**Table 3:** The half‐maximum inhibitory concentrations (IC50) of a number of commercially available chemotherapeutic medications and etodolac derivatives, as reported in the literature.

Compound	Time (h)		IC50 (µM)	
Etodolac	72	Leukemia	50−100	Nakamura et al. (2004) [60]
	24	SSC‐9 skin cancer	982.75	Abo Aasy et al. (2019) [62]
Etodolac R‐enantiomer SDX‐101	48	Multiple myeloma	490	Yasui et al. (2007) [63]
Etodolac analog SDX‐308	48	Multiple myeloma	23.8	Yasui et al. (2007) [63]
Celecoxib	24	MCF‐7	89.05	Huang et al. (2017) [64]
		MDA‐MB‐231	40.05	
Gefitinib	24	MCF‐7	11.27	Garduño‐Villavicencio et al. (2024) [65]
		MDA‐MB‐231	17.24	
Oxaliplatin	24	MCF‐7	7.4	Raymond et al. (1997) [66]
		MDA‐MB‐231	17.9	
Ibuprofen	48	MCF‐7	92.14	Daniel Pedro‐Hernández et al. (2020) [67]
5‐FU	24	MCF‐7	17.02	Badawi et al. (2023) [68]
		MDA‐MB‐231	11.73	

However, the main advantage here is that producing effective alternatives to etodolac, which is already in use as a well‐tolerated drug, could provide a faster improvement in the physiological responses and reduction of the side effects.

Whether or not NSAIDs' anticancer effects are exclusively attributable to COX‐2 inhibition has to be fully established, but research has shown that celecoxib, the most popular selective COX‐2 inhibitor in the market, demonstrates potent anti‐tumor activity via apoptosis, regulation of tumor microenvironment, or resensitization to other chemotherapeutic drugs. The effective range of cytotoxicity IC50 values of celecoxib in inhibiting cancer cell proliferation was reported as 25−100 µM [69]. The higher safety, lower toxicity, and effectiveness of celecoxib relative to the other available COX‐2 inhibitors urge it as a preferable chemopreventive drug. However, due to the drug's solubility issues, its dose–response relationship is compromised, which may increase cardiovascular risks based on the patient's medical history [70].

### 3.3 Assessment of Cell Death Induced by SGK 206 and SGK 217 Activity

**Figure 4:**
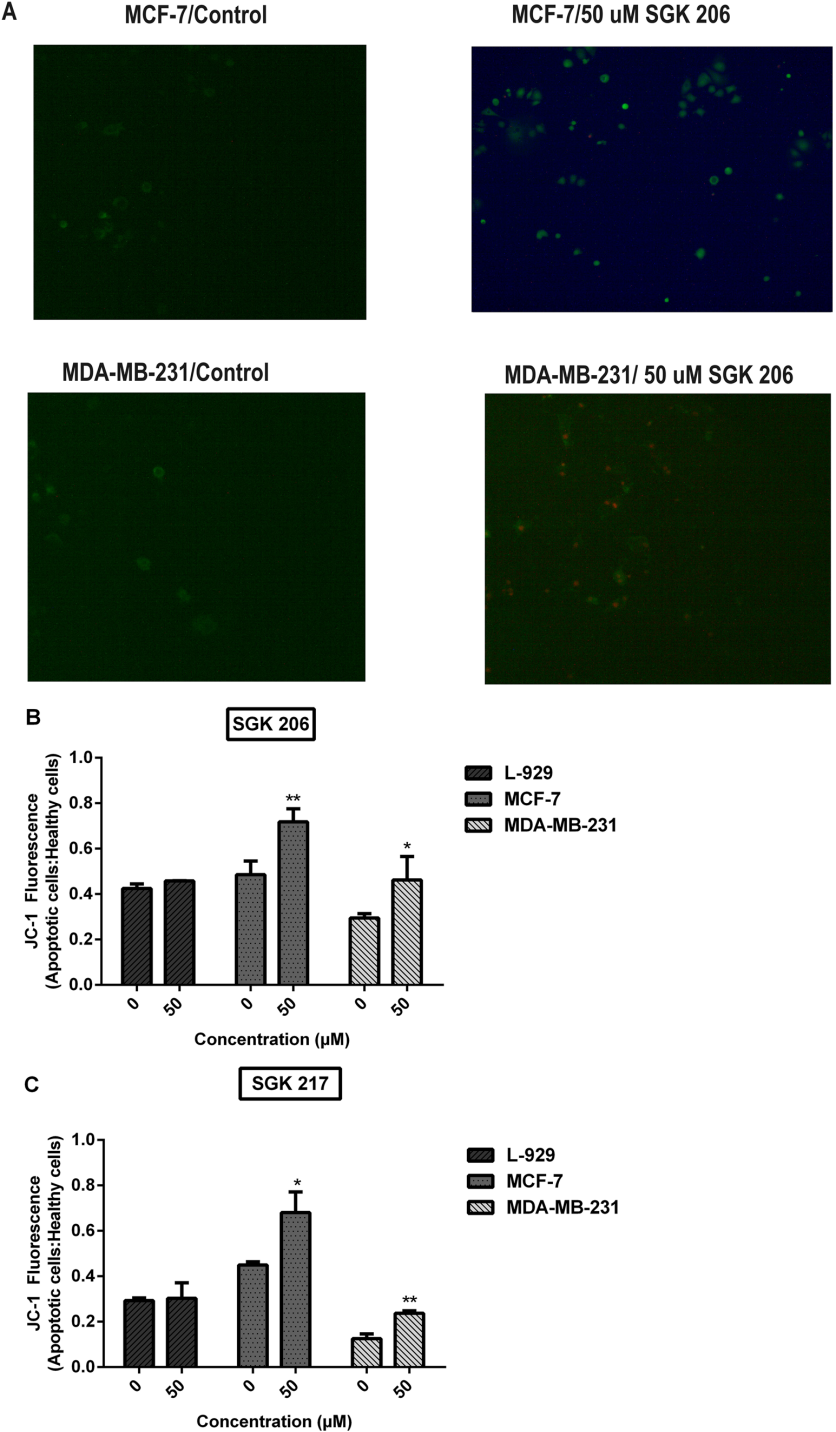
(A) Annexin V/PI staining of cells treated with SGK 206. The control group represents cells without any agent. The MMP test quantified the amount of apoptosis in (B) SGK 206 and (C) SGK 217 (0‐50 µM) after a 24‐h exposure. (*p < 0.05, **p < 0.01, ***p < 0.001, ****p < 0.0001).

To assess whether our compounds produce their cytotoxic effects through induction of apoptosis, apoptosis was investigated using Annexin V/PI labeling and MMP methods (Figures 4A−C). In addition, by utilizing TALI image‐based cytometry and fluorescent microscopy, the number of cells was analyzed for different concentrations of agents (Table 4).

**Table 4:** Percent (%) values of living, dead and apoptotic cells after 24 h of incubation with etodolac, SGK 206, and SGK 217 in MCF‐7 and MDA‐MB‐231 cells. Data are representative of three independent experiments.

Etod	Etod L/D/A (%)	SGK 206 SGK 217	SGK 206 L/D/A (%)	SGK 217 L/D/A (%)
MCF‐7				
0 µM	98/2/0	0 µM	95/5/0	95/3/0
100 µM	94/5/1	25 µM	89/9/2	87/7/6
250 µM	92/6/2	50 µM	81/15/4	74/10/16
500 µM	84/9/7	75 µM	62/19/19	55/27/18
1000 µM	87/8/5	100 µM	n.d.	n.d.
MDA‐MB‐231				
0 µM	96/4/0	0 µM	94/6/0	94/6/0
100 µM	95/4/1	25 µM	97/2/1	93/7/0
250 µM	94/5/1	50 µM	93/3/4	66/11/23
500 µM	84/10/6	75 µM	67/25/8	n.d
1000 µM	76/13/11	100 µM	n.d.	n.d.

Abbreviations: A, apoptotic; D, dead; Etod, etodolac; L, live; n.d., not determined.

In MMP assays, apoptotic increases became detectable only after 0.5−1.0 mM for etodolac, in consistency with MTT data. These increases were at similar levels for all cells, including the control cell line L‐929. Our synthesized compounds, on the other hand, showed apoptotic effects at much lower doses (≥ 25 μM). There was a concentration‐dependent increase in apoptosis following SGK 206 or SGK 217 addition, in agreement with MTT viability tests. Upon the addition of SGK 206 at 50 μM concentration, apoptosis was shown to increase by 48%, 57%, and 8% in MCF‐7, MDA‐MB‐231, and L‐929 cells, respectively (Figure 4A). Same concentrations of SGK 206 induced larger changes following 48 h incubation, and the percent of apoptotic cells increased twofold (data not shown).

In the same way, all the cell lines were incubated with low concentrations (10, 25, and 50 µM) of SGK 217 for 24 h. The apoptotic effect was considerably higher in cancer cell lines in comparison with the healthy fibroblast cell line. The addition of 50 μM SGK 217 enhanced apoptosis by 51%, 85%, and 3% in MCF‐7, MDA‐MB‐231, and L‐929 cells, respectively, compared to the control group.

Based on these data, it became clear that etodolac derivatives have at least twice the capacity to inhibit proliferation of cancer cells compared to the control L‐929 cell line and were more effective than etodolac at much lower doses. Our third compound, the triazole derivative of etodolac (SGK 242), induced severe toxicity in both breast cancer cells and controls even at a 25 µM concentration. The response was not concentration‐dependent. Due to its high toxicity, no further study was conducted with this compound.

### 3.4 Changes in Apoptotic Protein Expressions and COX‐2 Levels

The BCL‐2 is a large protein family with a principal role in regulating cell death through interactions between pro‐ and anti‐apoptotic (Bak, Bak, Bcl‐XL, Bad, etc.) proteins. These proteins, in turn, change the permeability of the mitochondrial membrane and allow the release of various apoptotic factors. Higher Bax/Bcl‐2 ratios have a crucial regulatory role in controlling the apoptotic destiny of cells by decreasing cellular resistance to apoptotic stimuli and promoting cell death [71, 72, 73, 74].

Bax/Bcl‐2 ratio was examined in cancer cell lines that had been exposed to etodolac, SGK 206, and SGK 217 for 24 h (Figure 5A). The ratio significantly increased in both cell lines when treated with 500 µM etodolac or 50 µM of SGK 206 for 24 h (Figure 5B).

Figure 5Representative immunoblot image of Bax, Bcl‐2, PARP, and COX‐2 protein expressions with β‐actin in breast cancer cells treated with etodolac and SGK 206 at the end of 24 h incubation (A). The expression levels of each band were quantified with scanning densitometry and normalized to beta‐actin for Bax, Bcl‐2 (B), c‐PARP (C), and COX‐2 (D). (*p < 0.05, **p < 0.01, ***p < 0.001, ****p < 0.0001). Control groups represent cell lines without any agent. Each column represents the mean ± SD from three experiments.

**Figure:**
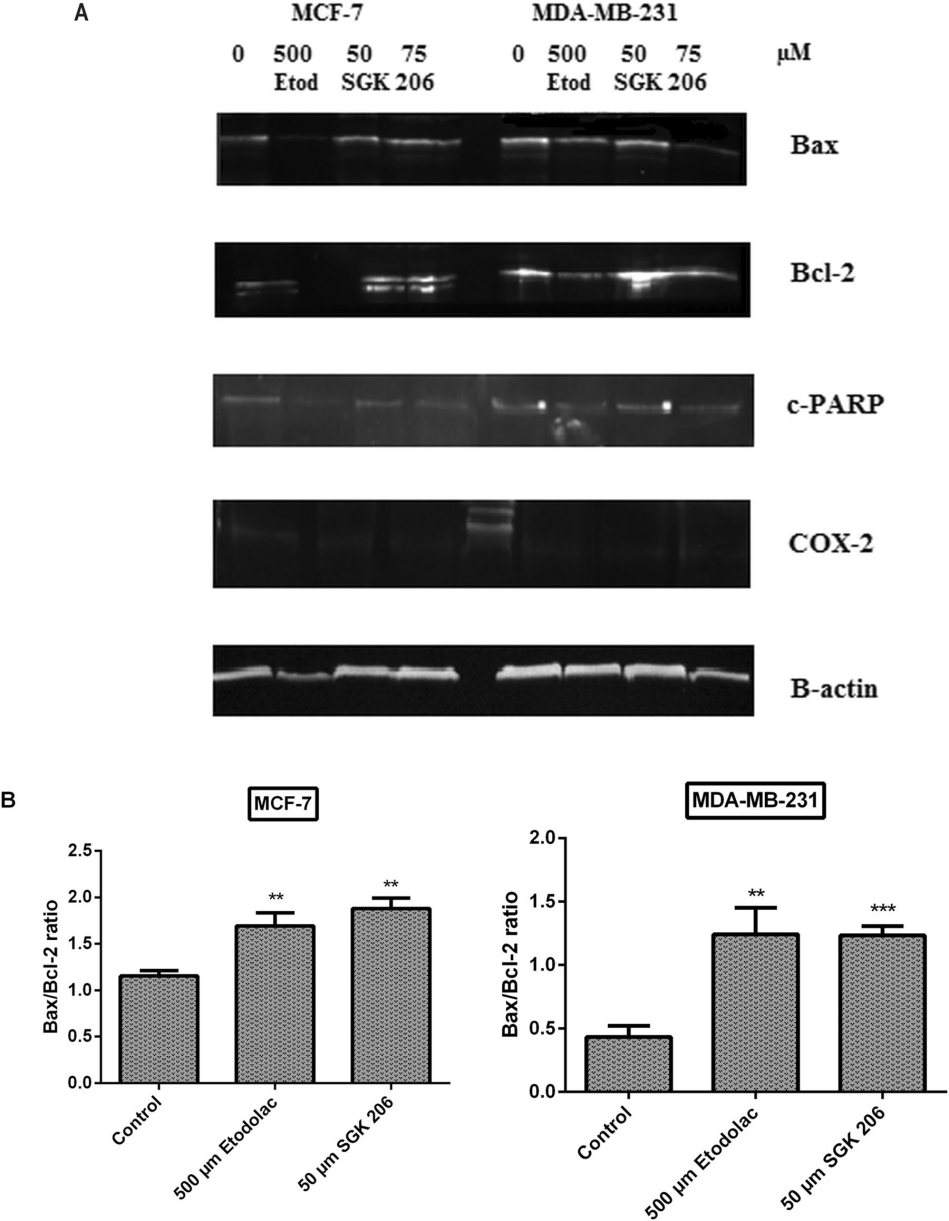

**Figure:**
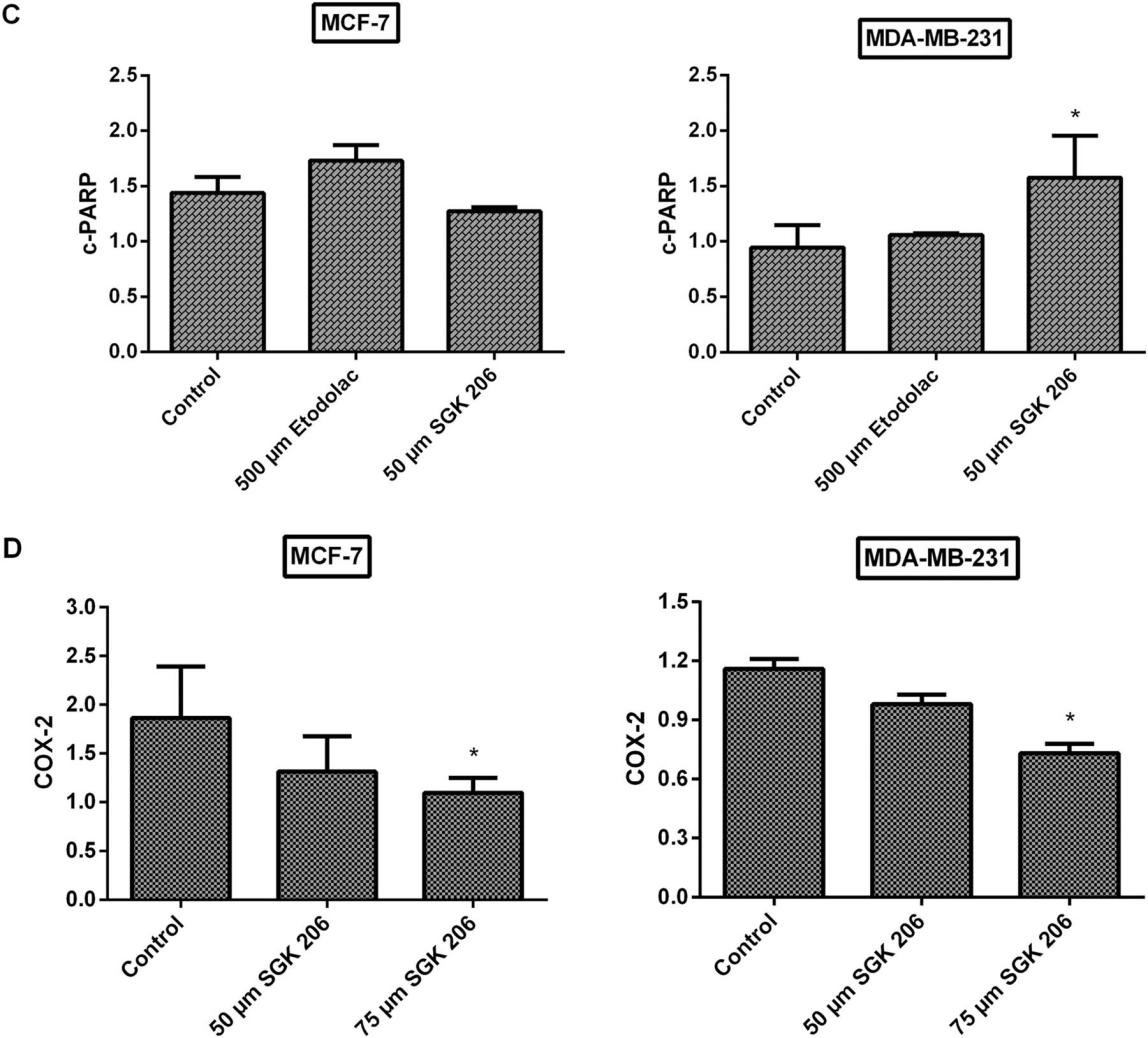

Cleavage of PARP‐1 is another hallmark of apoptosis. After the cleavage of PARP‐1 through caspase 3 or 7, two specific fragments are formed: an 89‐kD catalytic fragment and a 24‐kD death binding domain [75, 76]. The primary antibody was specific for the 89‐kD fragment in this study. After treatment with different concentrations of SGK 206 and SGK 217, a consistent activation was only observable in MDA‐MB‐231 cells for both derivatives (Figure 5C). It's possible that the absence of caspase‐3 activity in MCF‐7 cells kept us from seeing c‐PARP expression in this particular cell line [77, 78].

Immunoblot analyses were also performed in the presence of the synthetic substance SGK 217. The Bax/Bcl‐2 ratio was shown to significantly rise at two concentrations (50 and 75 µM) (Figure 6A,B). In PARP cleavage, measurable band intensity was again only obtainable in MDA‐MB‐231 cells. c‐PARP protein showed a significant increase at all concentrations in MDA‐MB‐231 cells (Figure 6C).

Figure 6Representative immunoblot image of Bax, Bcl‐2, PARP, and COX‐2 protein expressions with β‐actin in breast cancer cells treated with SGK 217 at the end of 24 h incubation (A). The expression levels of each band were quantified with scanning densitometry and normalized to beta‐actin for Bax, Bcl‐2 (B), PARP (C), and COX‐2 (D). (*p < 0.05, **p < 0.01, ***p < 0.001, ****p < 0.0001). Control groups represent cell lines without any agent. Each column represents the mean ± SD from three experiments.

**Figure:**
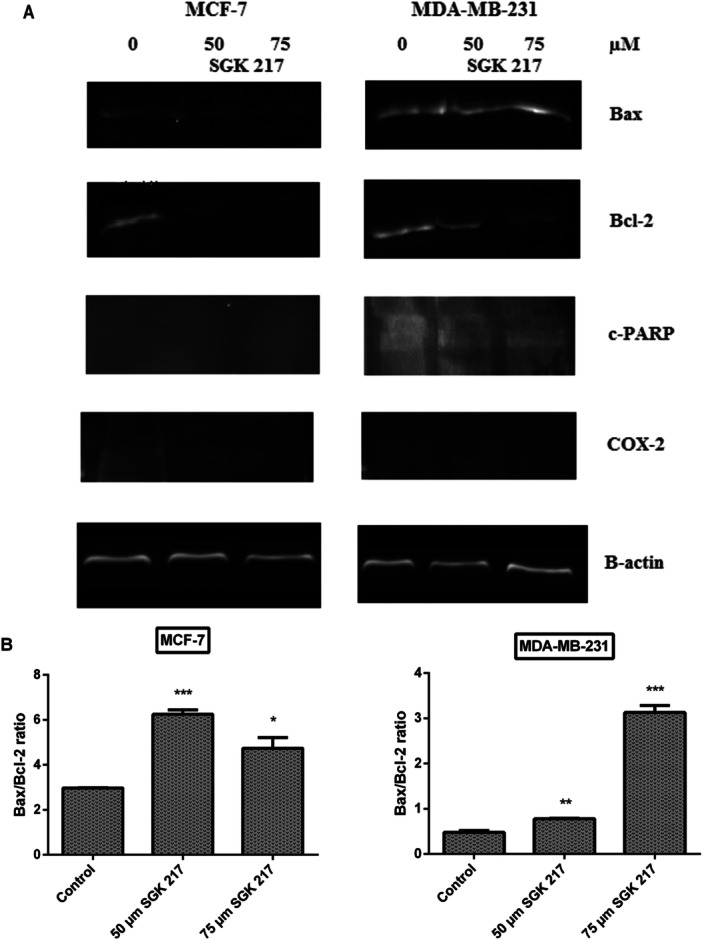

**Figure:**
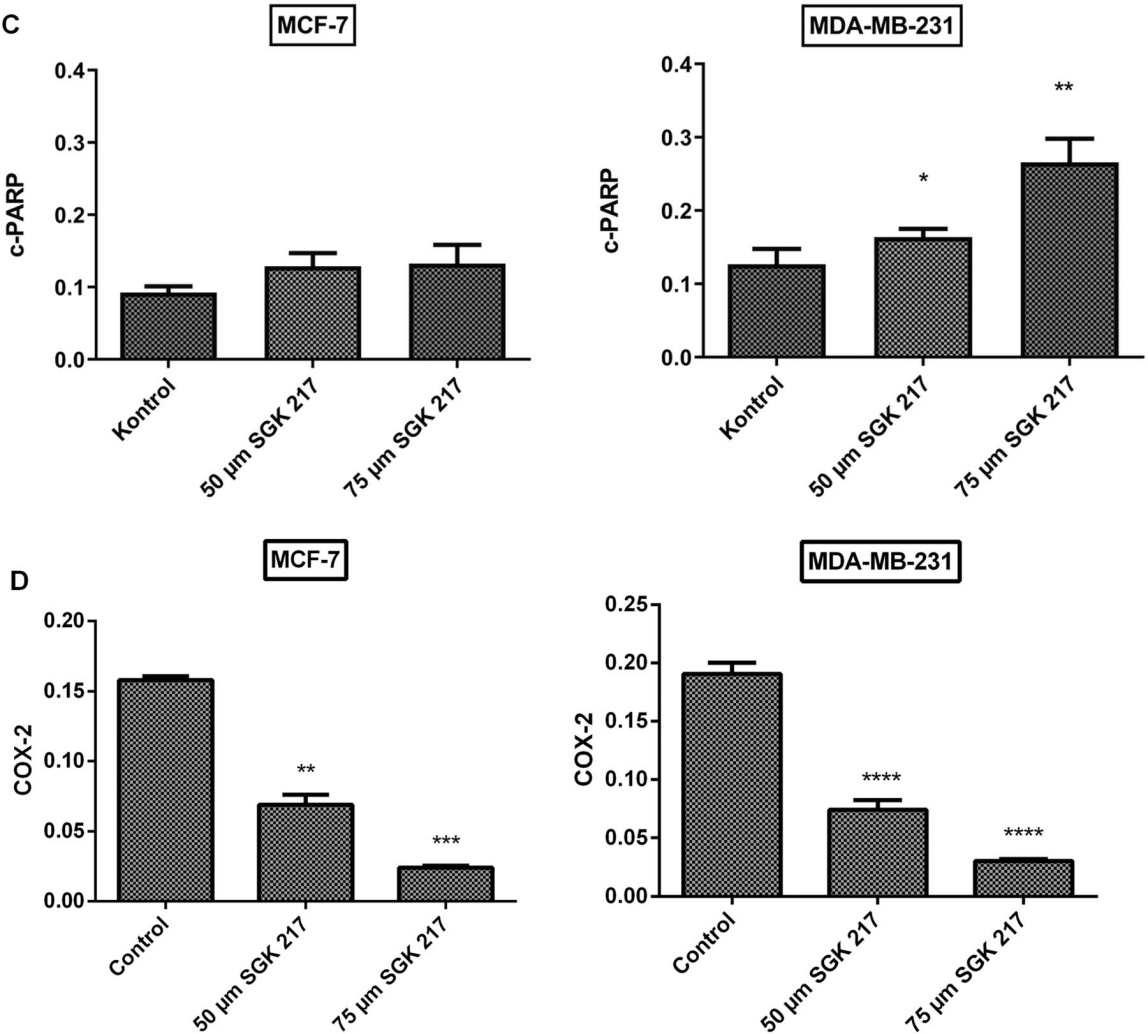

In addition to apoptosis‐related proteins, COX‐2 expression levels were also investigated for our potential COX‐2 inhibitors.

One of the earliest studies on the expression of COX‐1 and COX‐2 in breast cancer cell line cultures was a study conducted by Liu and Rose, who discovered that COX‐2 expression varied between hormone‐sensitive metastatic (MDA‐MB‐231) and weak‐metastatic cell lines (MCF‐7). The metastatic MDA‐MB‐231 cell line was shown to have significant COX‐2 activity, and besides, PGE2 synthesis was found to be well correlated with the level of COX‐2 protein [79].

Ottanà et al. looked into the inhibitory effects of a few 4‐thiazolidinone compounds on human colon cancer cell lines that expressed different amounts of COX‐2. The results of this study indicated that a 2‐phenylimino thiazolidinone derivative was a selective inhibitor of COX‐2 and inhibited the growth of HT‐29 cells, which express high levels of COX‐2 [80]. Similarly, results for many cancer cell lines were published on the impact of various hydrazone derivatives working through the COX‐2 pathway [81].

After 24 h of incubation with SGK 206, COX‐2 protein expression was downregulated in both breast cancer cell lines. At concentrations of 50 µM and 75 µM, COX‐2 protein expression was detected to decrease by 30% and 41% for MCF‐7 and 16% and 37% for MDA‐MB‐231, respectively, compared to the control group (Figure 5D). In the same way, SGK 217 displayed a significant decrease in COX‐2 protein expression in both cancer cell lines. At concentrations of 50 µM and 75 µM, COX‐2 protein expression levels were detected to decrease by 56% and 85% in MCF‐7 cells and 61% and 84% in MDA‐MB‐231 cells, respectively, compared to the control group (Figure 6D).

In line with these aforementioned studies, we reported here that hydrazone and the 4‐thiazolidinone derivative of etodolac decreased COX‐2 protein expression in both hormone‐receptor‐positive and triple‐negative breast cancer cell lines.

### 3.5 Effect of SGK 206 on PGE2 Release

Since an elevation in PGE2 levels through COX‐2 induction has been extensively reported in breast cancer cell lines, the mechanism of action in synthetic compounds was also investigated for PGE2 release [79, 80, 81, 82]. The experiments were only conducted with the hydrazone derivative SGK 206, which showed the best dose‐dependent response in breast cancer cells.

**Figure 7:**
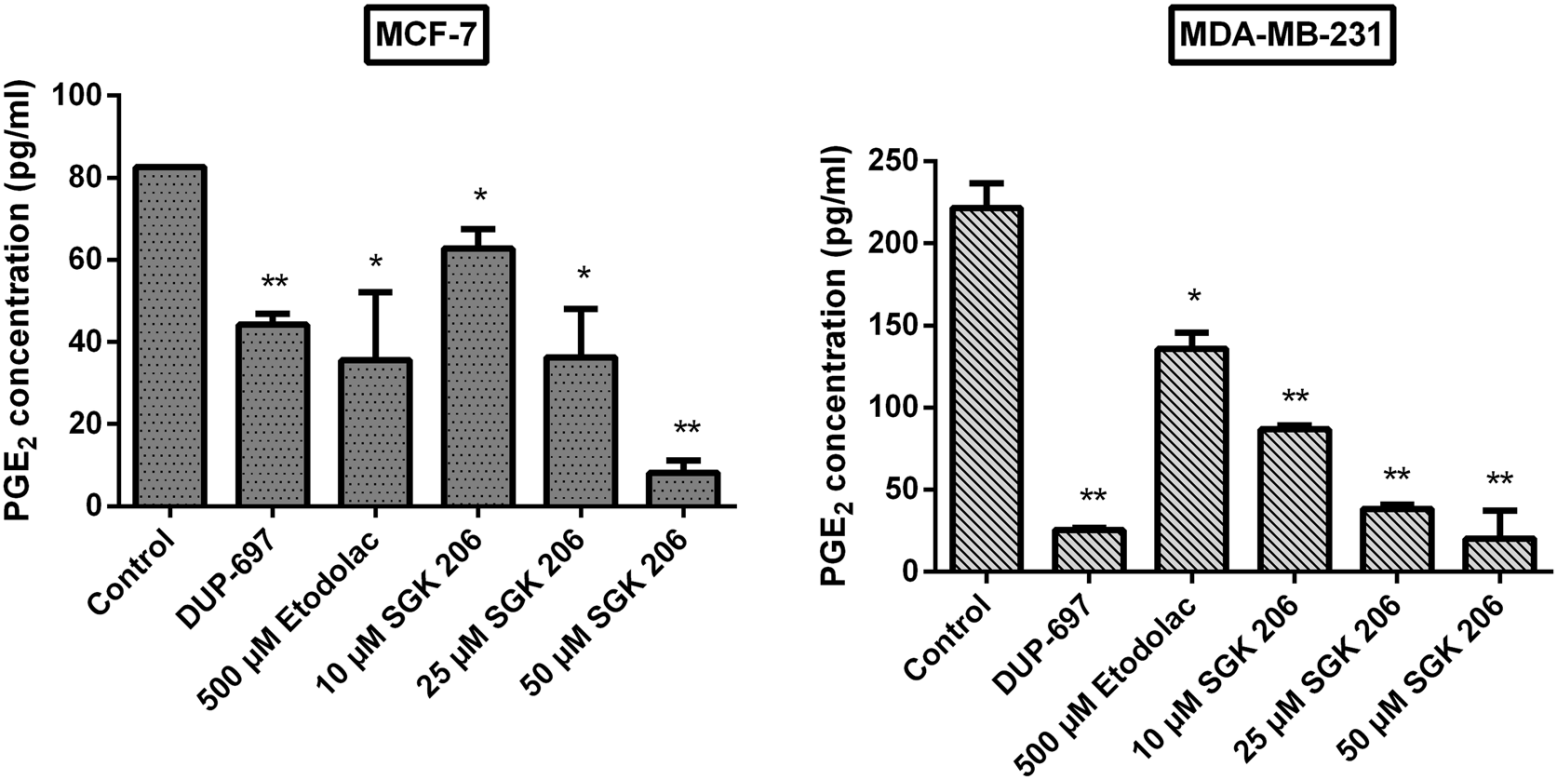
The PGE2 levels of MCF‐7 and MDA‐MB‐231 cells after 24 h incubation with a high concentration of etodolac and low concentrations of SGK 206 (3 µM DuP‐697, an active COX‐2 inhibitor, was employed as a positive control). (*p < 0.05, **p < 0.01, ***p < 0.001, ****p < 0.0001).

In comparison to the control group, SGK 206 dramatically reduced PGE2 release at all doses after 24 h of incubation. In comparison to the control groups, PGE2 levels of 0.5 mM etodolac and 50 µM SGK 206 inhibited PGE2 release by 57% and 90% in MCF‐7 and by 38% and 91% in MDA‐MB‐231, respectively (Figure 7). SGK 206 was more efficient than etodolac at much lower concentrations and produced significant inhibition in PGE2 levels of cancer cells, notably at 50 µM concentration. DUP‐697, on the other hand, is a well‐known agent for its strong and selective suppression of human prostaglandin G/H synthase [83, 84], reducing PGE2 release by 46% and 88% in MCF‐7 and MDA‐MB‐231, respectively, at a 3 µM dose.

Collectively, gathered data suggests that our derivatives may serve as etodolac substitutes due to their greater anti‐proliferative and apoptotic action. Although there were no appreciable changes between the two cell lines, the general response of MDA‐MB‐231 was more explicit compared to MCF‐7. This is to be expected given that MCF‐7 expresses higher levels of COX‐1 and a minor amount of COX‐2, whereas estrogen‐independent and highly aggressive type MDA‐MB‐231 expresses constitutive COX‐2 at high levels and produces five times as much PGE2 [79]. Furthermore, MDA‐MB‐231 cells exhibited larger quantities of apoptotic protein, which might be explained by the absence of caspase‐3 activity in MCF‐7 cells.

Initially developed as an anti‐inflammatory and anti‐nociceptive medication, etodolac was subsequently discovered to have anti‐cancer effects as well. However, because of the limited selectivity between COX‐1/COX‐2 actions, common long‐term or high‐dose usage of it has resulted in stomach or cardiovascular adverse effects [85, 86, 87, 88, 89].

The subsequent development of more specific COX‐2 inhibitors—NSAIDs like celecoxib, rofecoxib, and valdecoxib—which are also used as analgesics, anti‐inflammatories, and antirheumatic medications, resulted in deregulation of the coagulatory balance and therefore caused serious vasodilatory and clotting problems [90, 91].

Our synthesized compounds have the advantage of having higher effects in lower doses, which may minimize potential side effects, exhibit no detrimental toxicity on normal cells, induce apoptosis, and significantly suppress COX‐2 expression and PGE2 activity. We believe that these early findings could initiate more research on their anti‐inflammatory actions, signal mechanisms, and preclinical xenograft testing studies.

## 4 Conclusions

Based on these findings, both the etodolac hydrazone derivative, SGK 206, and the thiazolidinone derivative, SGK 217, were found to be more effective than etodolac in invasive (MDA‐MB‐231) and non‐invasive (MCF‐7) breast cancer cell types. The two etodolac derivatives were equally effective, but SGK 206 in particular demonstrated a distinct dose–response relationship, a lower IC50 value, and a stronger apoptotic impact, especially on MDA‐MB‐231 cells. This chemical has the potential to be a compelling novel therapeutic candidate that drives future investigation. We anticipate that these results will help guide future research on etodolac‐based treatments.

## Ethics Statement

Ethical approval for this study was not required because the research involved the use of established cell lines, which are commercially available. These cell lines do not involve human or animal subject research that requires ethical committee approval.

## Consent

The authors have nothing to report.

## Conflicts of Interest

The authors declare no conflicts of interest.

## Data Availability

The data supporting the findings of the article is available within the article.
